# Association of Anthropometric Indices with Menstrual Abnormality among Nursing Students of Nepal: A Cross-Sectional Study

**DOI:** 10.1155/2022/6755436

**Published:** 2022-03-18

**Authors:** Kapil Amgain, Prativa Subedi, Gopal Kumar Yadav, Sujana Neupane, Sitaram Khadka, Shubha Devi Sapkota

**Affiliations:** ^1^Department of Clinical Anatomy and Cell Biology, Karnali Academy of Health Sciences, Jumla, Nepal; ^2^Department of Emergency Medicine, Rolpa District Hospital, Rolpa, Nepal; ^3^Department of Emergency Medicine, Kalaiya District Hospital, Bara, Nepal; ^4^Department of Nursing, Manmohan Cardiothoracic Vascular and Transplant Center, Maharajgunj, Kathmandu, Nepal; ^5^Clinical Pharmacist and Pharmacologist, Shree Birendra Hospital, Nepalese Army Institute of Health Sciences, Kathmandu, Nepal; ^6^Narayani Polytechnic Institute, Narayani Samudayik Hospital, Chitwan, Nepal

## Abstract

**Introduction:**

Obesity has been reported to be linked with menstrual abnormalities including abnormality in cycle length, duration, and period blood loss. However, which anthropometric parameter is a better marker of menstrual abnormality is yet unknown. This study aims to explore the association of BMI, waist-hip ratio (WHR), and waist-height ratio (WHtR) with menstrual abnormalities.

**Methods:**

This was a cross-sectional study conducted among 240 nursing students on two nursing campuses of Nepal. Demographic and menstrual characteristics related data were collected from the participants via the face-to-face interview technique followed by anthropometric measurements. Binary logistic regression was used to study the association of BMI, WHR, and WHtR with menstrual characteristics. Univariable and multivariable regression models were calculated at 95% confidence interval and a *P* value of 0.05 using a Statistical Package for Social Sciences, IBM SPSS® v21 (IBM, Armonk, New York).

**Results:**

Out of 240 participants, 52 participants (21.67%) were underweight (<18.5 kg/m^2^), and 47 participants (19.58%) were either overweight (≥23 kg/m^2^) or obese (≥25 kg/m^2^). Overweight and obese BMI was associated with abnormality in menstrual cycle length (AOR = 4.24; 95% CI = 1.77–10.17), duration of the menstrual period (AOR = 2.52; 95% CI = 1.09–5.81), and periodic menstrual blood loss (AOR = 9.04; 95% CI = 3.55–23.01). Increase in WHtR (>0.5) was associated with abnormal cycle length (AOR = 3.09; 95% CI = 1.09–8.80) and abnormal period duration (AOR = 3.05; 95% CI = 1.10–8.44). An increase in WHR (>0.8) was associated with abnormal cyclical menstrual blood loss (AOR = 4.93; 95% CI = 1.55–15.71).

**Conclusions:**

Obesity predisposes to menstrual irregularities. BMI is a better predictor of menstrual irregularity as the increase in BMI is associated with abnormality in menstrual cycle length, period duration, and blood loss. Increased WHR was associated with periodic blood loss only. Increased WHtR was associated with abnormal cycle length and period duration, but not menstrual blood loss.

## 1. Introduction

Anthropometric indices encompass quantitative measurements of the muscle, bone, and adipose tissue and are used to assess body composition and nutritional status of an individual [1]. Anthropometric parameters viz. height, weight, and body circumferences (waist and hip) have been used to calculate the anthropometric indices such as body mass index (BMI), waist-to-hip ratio (WHR), and waist-to-height ratio (WHtR). There are variations in body fat content according to age, sex, race, ethnicity, and geography. Studies show that obesity leads to a myriad of complications including cardiovascular and cerebrovascular diseases, gastroesophageal reflux, sleep disorders, dementia, and even depression [2]. Obesity is also linked with infertility, abnormal menstrual cycle, and even endometrial carcinoma [3, 4].

Menstruation is a physiological process characterized by cyclical bleeding per vagina due to the shedding of the endometrial lining following a decline in the level of estrogen and progesterone hormones. A normal menstrual cycle occurs in the interval of 21–35 days, with a cycle duration of 3–7 days, and blood loss of less than 80 ml [5, 6]. A normal menstrual cycle indicates an intact hypothalamus-pituitary ovarian axis. It reflects a state of hormonal balance and sound reproductive capacity [7]. The menstrual cycle is affected by several factors including smoking, alcohol, stress, physical activity, nutritional status, ethnicity, and body build [8, 9]. A study among high school girls showed that menstrual characteristics were significantly affected by height, weight, BMI, and body circumferences [10]. This study aims to check the association of menstrual abnormality with BMI, WHR, and WHtR among nursing students of Nepal and also aims to explore which anthropometric parameter is a better predictor of menstrual abnormality.

## 2. Materials and Methods

### 2.1. Setting

Nepal is a landlocked developing country situated in Southeast Asia. It ranks 142 out of 189 countries in terms of the human development index (HDI) and has an HDI of 0.602 in 2019 [11]. With an area of 1,47,1516 sq. km, Nepal is divided into 7 federal provinces, 77 districts, and consists of 3 principal physiographic belts: Himalaya, Hilly, and Terai regions.

### 2.2. Research Design and Participants

**Figure 1:**
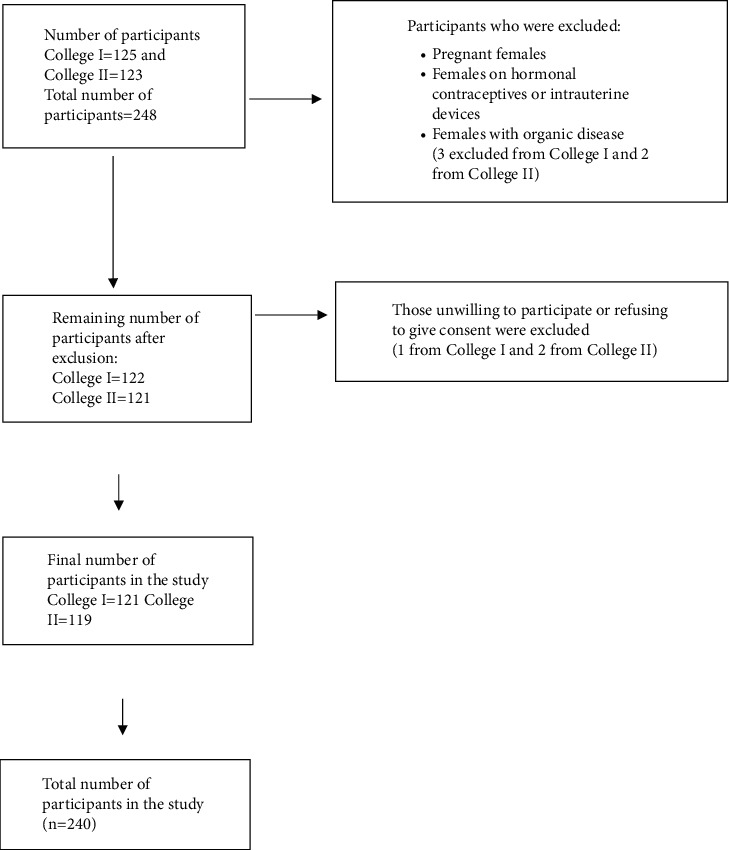
Flow diagram showing the selection of participants.

This was a cross-sectional study conducted from July 2019 to October 2019 in two nursing campuses, one each of Karnali province and Bagmati province of Nepal. These two campuses were purposively selected. All the female nursing students with ages ranging from 16 to 24 years who have had their menarche and consented to participate were included in this study. The participants who were pregnant, using hormonal contraceptives or intrauterine devices, and with any known organic diseases and/or under any medication were excluded from this study. Participants who were missing during the first visit were covered during the next visit within a week to increase the participation rate in the study. A total of 243 participants met the inclusion criteria. However, three participants refused to participate in the study. Thus, the final sample size was 240.  Figure 1 shows the flow diagram of the selection of participants.

### 2.3. Data Acquisition

Data were collected from each participant by the face-to-face interview technique. The standard pretested and semistructured questionnaire was used to collect the data related to current age in completed years, age at menarche (in completed years), and menstrual characteristics including length of the menstrual cycle, duration of the menstrual period, and the number of pads used per menstrual period to quantify the periodic menstrual blood loss. After the face-to-face interview, anthropometric measurements were taken. Height, weight, hip circumference, and waist circumference were measured by the same investigators to avoid interpersonal bias during the measurements as per the criteria defined by the WHO [12].

### 2.4. Assessment of Anthropometric Parameters

Asia-Pacific classification of BMI by Western Pacific Regional Office was considered for categorization of BMI in our study [13]. The cutoff range for normal BMI is considered 18.5–22.9 kg/m^2^ with BMI <18.5 kg/m^2^ being regarded as underweight, BMI of 23–24.9 kg/m^2^ as overweight, and BMI of ≥25 kg/m^2^ as obesity. Likewise, WHR less than 0.8 and WHtR less than 0.5 are considered normal for women, and levels above them were regarded as high [14].

All the anthropometric indices were calculated as per FANTA guidelines [14]. All measurements were taken with minimum clothes. The weight and height were taken without shoes, and hip and waist circumferences were measured in anatomical position at the end of normal expiration. The weight was measured to the nearest 0.1 kilograms (kg) using a digital weighing machine (Nureca Inc., NY, USA). Height was measured in centimeters with the help of a stadiometer (Seca, Hamburg, Germany) with a precision of 0.1 centimeters (cm). The waist circumference and hip circumference were measured in centimeters at the nearest 0.1 cm by using an inelastic measuring tape (SellnShip Tailor Inch Tape, India).

BMI was calculated as weight in kilograms divided by the square of the height of the participant in meters. WHR was calculated by dividing the waist circumference by the hip circumference of the participant, and WHtR was calculated by dividing the waist circumference by the height of the participant.

### 2.5. Assessment of Menstrual Characteristics

Participants were asked about the characteristics of their menstrual cycle based on their recall of the characteristics of the past three months. The length of the menstrual cycle was defined as the period between the first day of two consecutive menstruations, while the duration of the menstrual period was defined as the days of menstrual bleeding per menstrual cycle.

There is a lot of discrepancy in regards to the normal length of the menstrual cycle, duration of the menstrual period, and amount of blood loss during menstruation. We had conducted an initial pretest among 25 students of a nursing college. Based on the results of the pretest and previous literature, we have considered the normal length of the menstrual cycle as 21–35 days and the normal duration of the menstrual period as 3–7 days [5, 15]. During our pretest, the nursing students mentioned that they changed their pads at least 3-4 times a day, even if they were partially soaked. Thus, relating it to the normal duration of the menstrual period, we have considered 15–20 pads as moderate or normal menstrual bleed. Pads less than 15 and pads more than 20 were considered as mild and heavy bleeding, respectively, and were categorized into abnormal menstrual blood loss.

### 2.6. Data Management and Statistical Analysis

The data from the questionnaire and proforma were first thoroughly checked, coded, and entered into Microsoft Excel 2019 v16.0 (Microsoft, WA, USA). It was then transferred into a Statistical Package for Social Sciences, IBM SPSS® v21 (IBM, Armonk, New York) for the statistical calculations. Demographic, anthropometric, and menstrual characteristics of study participants were presented in the table as frequency, proportions, mean ± SD, and range. The chi-square test was used to test for group differences. For univariable logistic regression analyses, odds ratios (OR) and 95% confidence interval (CI) were calculated. Multivariable logistic regression was used to determine independent anthropometric parameters associated with menstrual characteristics, and adjusted odds ratios (AOR) were calculated at 95% confidence interval (CI). All variables with *P* < 0.20 were retained in the final multivariable model.

### 2.7. Ethical Approval

The aims and objectives, data collection procedure, possible risks, and benefits of participating in this study were well explained to the participants, and they were assured that their participation in the study was voluntary and they may quit at any stage of filling the questionnaire. Both written and verbal informed consent were taken before the study. All procedures were approved by Nepal Health Research Council (reference no. 890) and were conducted under the Declaration of Helsinki for human studies of the World Medical Association.

## 3. Results

### 3.1. Demographic, Anthropometric, and Menstrual Characteristics of Study Participants

Out of 240 participants, most of them belonged to the age group ≥20 years (161, 67.08%), and a majority of them (155, 64.58%) had their menarche at age 11–14 years. Most of the participants had normal BMI (141, 58.75%), followed by underweight (52, 21.67%), overweight (35, 14.58%), and obese (12, 5.00%). Waist-to-hip ratio (WHR) and waist-to-height ratio (WHtR) were normal in about half of the participants (133, 55.41% and 155, 64.58%) (Table 1).

The length of the menstrual cycle was normal in three-fifths (146, 60.83%) followed by oligomenorrhea (56, 23.34%) and polymenorrhagia (38, 15.83%). Duration of the menstrual period was normal in only about half of the participants (125, 52.50%), while periodic blood loss was normal in about two-thirds of the participants (156, 65.00%) (Table 2).

### 3.2. Association of Anthropometric Parameters with Menstrual Characteristics

Participants with overweight and obese BMI had 4.24 odds of having an abnormal menstrual cycle length compared to normal BMI (AOR = 4.24, 95% CI = 1.77–10.17, *P* value = <0.001). Participants with underweight BMI had 2.08 odds of having an abnormal menstrual cycle length. However, the association was not statistically significant (AOR = 2.08, CI = 0.88–4.92, *P* value = 0.097). Length of menstrual cycle was not associated with WHR but was positively associated with WHtR (AOR = 3.09, 95% CI = 1.09–8.80, *P* value = 0.034) (Table 3).

Abnormal duration of the menstrual period was seen in participants with increased BMI (AOR = 2.52, 95% CI = 1.09–5.81, *P* value = 0.030) and increased WHtR (AOR = 3.05, 95% CI = 1.10–8.44, *P* value = 0.032). Although underweight individuals had 1.35 odds of having abnormal duration of the menstruation period, the association was not statistically significant. (AOR = 1.35, CI = 0.59–3.08, *P*value = 0.482) (Table 4).

Periodic menstrual blood loss was higher in underweight (AOR = 4.42, 95% CI = 1.79–10.92, *P* value = 0.001) as well overweight and obese individuals (AOR = 9.04, CI = 3.55–23.01, *P* value = <0.001) as compared to participants with normal BMI. Menstrual blood loss was not associated with WHtR but was significantly associated with WHR (AOR = 4.93, CI = 1.55–15.71, *P* value = 0.007) (Table 5).

## 4. Discussion

The average BMI of participants in our study was 20.3 ± 3.1 kg/m^2^. A cross-sectional study conducted in 2021 in Nepal showed that the average BMI of the overall population was 20.9 ± 1.8 kg/m^2^ [16]. Likewise, another descriptive cross-sectional study conducted among the medical students of Nepal showed that the average BMI was 20.8 ± 2.4 kg/m^2^ [17].

In our study, the prevalence of overweight and obesity was 14.58% and 5.00%, respectively, which was lower as compared to other studies. Results from Nepal Demographic Health Survey, NDHS 2016, showed that the prevalence of overweight and obesity in Nepalese men and women was 28.77% and 32.87%, respectively, and the prevalence of underweight was 17.27% [18]. Although a large cutoff has been considered for obesity in the NDHS study (≥25 kg/m^2^), the higher prevalence of obesity in this study could be because this study was done among individuals above the age of above 50. Since this study comprised more of the adult population, the prevalence of obesity was higher. In a study done among adolescent girls in Bharatpur Metropolitan City, 23.90% were overweight and 10.40% were obese [19]. The lower prevalence of overweight and obesity in our study could be because our study was done among young girls only, while the NDHS survey includes the adult population. The prevalence of obesity increases with age with adults being at a higher risk compared to young people. This could be partly because insulin resistance increases with age [20]. On the other hand, the higher prevalence of overweight and obesity in the adolescents-based study done in Bharatpur could be due to variation in geography, climate, and ethnicity. Another pertinent reason for the difference could be that this study was done among nursing students. Healthier individuals are expected into nursing programs. Furthermore, we have excluded students with medical problems or with those who use chronic medications, likely excluding some of the heavier women (excluded based on hypertension, diabetes, and hypothyroid). In our study, the proportion of participants with abnormal menstrual cycle length, abnormal menstrual period duration, and abnormal periodic menstrual bleeding was 39.17%, 47.50%, and 35.00%, respectively. A study done among adolescent girls in Pokhara, Nepal, showed that the proportion of girls with abnormal menstrual cycle was relatively lower with 25.77%, while those with abnormal period duration was 11.15% [21]. Likewise, in a study by Nazish et al., among young female students studying health sciences, the proportion of participants with the abnormal menstrual cycle, abnormal menstrual period, and abnormal periodic menstrual bleeding was 21.60%, 29.20%, and 34.20%, respectively [22]. In a study by Karout et al., among female nursing students, the proportion of participants with the abnormal menstrual cycle and abnormal menstrual period was 59.49% and 13.59% [23]. Thus, the pattern of the menstrual cycle was similar in most of the studies within and outside Nepal.

Our study showed that menstrual cycle abnormalities including abnormal cycle length, period duration, and periodic blood loss was higher in overweight and obese individuals as opposed to those with normal BMI. Adipose tissue in obese individuals increases the conversion of androgen into estrogen. Furthermore, the decreased level of sex hormone-binding globulin in obese individuals leads to higher free estradiol levels [24]. The raised estrogen level causes thickening of the endometrial lining, thus contributing to heavy and prolonged menstrual bleeding. A higher incidence of menstrual irregularity in overweight and obese females can also be attributed to the higher incidence of anovulatory cycles in them [25]. Polycystic ovarian syndrome (PCOS) which is a common endocrinopathy in women of the reproductive age group is usually seen in obese females, and irregular menstruation is one of the major presenting complaints. The linear relation between obesity and menstrual irregularity in PCOS is supported by the fact that increased weight exacerbates the clinical features of PCOS including menstrual irregularity while weight loss results in significant improvement [26, 27].

Although there has been much research in regards to the relation of menstrual irregularity and menstrual blood loss with weight and BMI, only a few studies have incorporated indices like WHR and WHtR. While BMI reflects overall adiposity, WHR and WHtR are considered surrogate markers for central adiposity. Central obesity is found to be more closely linked with the complications of metabolic syndrome, normal-weight central obesity being more deadly than mere high BMI [28]. In regards to the menstrual cycle, a study by Lakshmanan et al. among women of age 18–40 showed that waist-hip ratio was a better indicator of menstrual irregularity than BMI [29]. However, in our study, WHR did not show a significant association with other parameters of menstruation except periodic menstrual blood loss. WHtR, on the other hand, did not affect periodic menstrual blood loss but was positively associated with length of menstrual cycle and duration of a menstrual period. It is unclear why WHR and WHtR showed less causation with menstrual abnormality than BMI.

Our study showed that being underweight did not pose the risk for abnormal menstrual cycle length and duration. However, both extremes of BMI were associated with abnormal periodic blood loss compared to individuals with normal BMI, although the risk of abnormal periodic menstrual bleeding was higher in overweight and obese compared to underweight individuals. In the study by Yunhui Tang, the odds of having heavy menstrual bleeding were less in underweight individuals as compared to those with normal BMI [30]. Increased menstrual blood loss in obese women is attributed to delayed endometrial repair secondary to a proinflammatory local endometrial environment in obese women [31]. Likewise, delayed endometrial repair in underweight females could be liked with the role of nutrition in healing. There is a paucity of literature reporting the association of abnormal menstrual bleeding with BMI.

We used logistic regression to study the strength of association of menstrual characteristics with BMI, WHR, and WHtR. The inclusion of participants from two different provinces with varied geographical settings helped to minimize the bias of climate and geography in menstrual characteristics. A face-to-face interview technique was used in our study, which increased the authenticity of our data. Our study had also certain limitations. We did not take into account other characteristics such as pain during menstruation, menstruation-related symptoms, physical activity, food habits, and perceived stress. We did not study the association of age of participants and their menarcheal age with menstrual characteristics.

## 5. Conclusion

Being overweight and obese had a positive association with menstrual abnormalities. BMI is a better predictor of menstrual irregularity as increased BMI was associated with menstrual abnormality in terms of cycle length, period duration, and periodic menstrual blood loss. Increased WHR was associated with periodic blood loss only. Increased WHtR was associated with abnormal cycle length and period duration but not with periodic menstrual blood loss. Thus, weight control is important in women for regular and healthy menstruation.

## Figures and Tables

**Table 1:** Demographic and anthropometric characteristics of study participants (*N* = 240).

S. no.	Demographic and anthropometric characteristics		Frequency	Proportion (%)
1	Age group (mean ± SD = 19.2 ± 2.5), range (16–24)	<20 years	79	32.92
		≥20 years	161	67.08

2	Age of menarche (mean ± SD = 13.3 ± 1.5), range (7–19)	≤10 years	41	17.08
		11–14 years	155	64.58
		≥15 years	44	18.34

3	BMI (mean ± SD = 20.9 ± 3.1), range (14.5–32.46)	Normal	141	58.75
		Underweight	52	21.67
		Over weight	35	14.58
		Obese	12	5.00

4	Waist-hip ratio (WHR) (mean ± SD = 0.9 ± 0.3), range (0.65–1.24)	Normal (≤0.8)	133	55.41
		High (>0.8)	107	44.59

5	Weight-height ratio (WHtR) (mean ± SD = 0.5 ± 0.3), range (0.28–0.63)	Normal (≤0.5)	155	64.58
		High (>0.5)	85	35.42

**Table 2:** Menstrual characteristics of study participants.

Menstrual characteristics of the participants		Frequency	Proportion (%)
Length of the menstrual cycle	<21 days (polymenorrhea)	38	15.83
	21–35 days (normal)	146	60.83
	>35 days (oligomenorrhea)	56	23.34

Duration of the menstrual period	<3 days	65	27.08
	3–7 days	126	52.50
	>7 days	49	20.42

Periodic menstrual blood loss^1^	Mild (<15 pads)	59	24.58
	Moderate (15–20 pads)	156	65.00
	Heavy (>20 pads)	25	10.42

^1^Moderate category of periodic menstrual blood loss was taken as normal and the rest were categorized as abnormal.

**Table 3:** Relation of anthropometric parameters with the length of the menstrual cycle by using binary logistic regression analysis.

Anthropometric parameters	Normal cycle^1^ (*n* = 146) (no. (%))	Abnormal cycle^2^ (*n* = 94) (no. (%))	Univariable model			Multivariable model^#^		
			Odds ratio (OR)	95% CI	*P* value	Adjusted OR	95% CI	*P* value
BMI					<0.001			
Normal (*n* = 141)	113 (80.14)	28 (19.86)	1			1.0		
Underweight (*n* = 52)	19 (36.54)	33 (63.46)	7.01	3.48–14.11	<0.001	2.08	0.88–4.92	0.097
Overweight and obese (*n* = 47)	14 (29.79)	33 (70.21)	9.51	4.49–20.13	<0.001	4.24	1.77–10.17	0.001
WHR					<0.001			
Normal (*n* = 133)	112 (84.21)	21 (15.79)	1			1.0		
High (*n* = 107)	34 (31.8)	73 (68.2)	11.45	6.17–21.26	<0.001	2.78	0.96–8.05	0.060
WHtR					<0.001			
Normal (*n* = 155)	123 (79.35)	32 (20.65)	1			1.0		
High (*n* = 85)	23 (27.05)	62 (72.94)	10.36	5.59–19.19	<0.001	3.09	1.09–8.80	0.034

^1^Normal length of menstrual cycle = 21–35 days of menstrual cycle. ^2^Abnormal length of menstrual cycle = less than 21 and more than 35 days of menstrual cycle. ^#^Adjusted for BMI, WHR, and WHtR.

**Table 4:** Relation of anthropometric parameters with duration of the menstrual period by using binary logistic regression analysis.

Anthropometric parameters	Normal period^1^ (*n* = 126) (no. (%))	Abnormal period (*n* = 114) (no. (%))	Univariable model			Multivariable model^#^		
			Odds ratio (OR)	95% CI	*P* value	Adjusted OR	95% CI	*P* value
BMI					<0.001			
Normal (*n* = 141)	93 (65.96)	48 (34.04)	1			1.0		
Underweight (*n* = 52)	19 (36.54)	33 (63.46)	0.8	0.6–1.6	<0.001	1.35	0.59–3.08	0.482
Overweight and obese (*n* = 47)	14 (29.79)	33 (70.21)	1.1	0.8–1.5	<0.001	2.52	1.09–5.81	0.030
WHR					<0.001			
Normal (*n* = 133)	92 (69.17)	41 (30.83)	1			1.0		
High (107)	34 (31.78)	73 (68.22)	4.82	2.78–8.34	<0.001	1.47	0.54–4.05	0.453
WHtR					<0.001			
Normal (*n* = 155)	103 (66.45)	52 (33.55)	1			1.0		
High (*n* = 85)	23 (27.06)	62 (72.94)	5.34	2.98–9.57	<0.001	3.05	1.10–8.44	0.032

^1^Normal duration of the menstrual period = 3–7 days of menstrual period. ^2^Abnormal duration of menstrual period = less than 3 and more than 7 days of the menstrual period. ^#^Adjusted for BMI, WHR, and WHtR.

**Table 5:** Association of anthropometric parameters with periodic menstrual blood loss (defined by no. of menstrual pads/cycle) by using binary logistic regression analysis.

Anthropometric parameters	Normal bleeding^1^ (*n* = 156) (no. (%))	Abnormal bleeding^2^ (*n* = 84) (no. (%))	Univariable model			Multivariable model^#^		
			Odds ratio (OR)	95% CI	*P* value	Adjusted OR	95% CI	*P* value
BMI					<0.001			
Normal (*n* = 141)	125 (88.65)	16 (11.35)	1 (Ref.)			1.0		
Underweight (*n* = 52)	18 (34.62)	34 (65.38)	14.76	6.81–31.96	<0.001	4.42	1.79–10.92	0.001
Overweight and obese (*n* = 47)	13 (27.66)	34 (72.34)	20.43	8.96–46.59	<0.001	9.04	3.55–23.01	<0.001
WHR					<0.001			
Normal (*n* = 133)	121 (90.98)	12 (9.02)	1 (Ref.)			1.0		
High (107)	35 (32.71)	72 (67.29)	20.74	10.12–42.51	<0.001	4.93	1.55–15.71	0.007
WHtR					<0.001			
Normal (*n* = 155)	131 (84.52)	24 (15.48)	1 (Ref.)			1.0		
High (*n* = 85)	25 (29.41)	60 (70.59)	13.10	6.92–24.79	<0.001	2.22	0.74–6.66	0.154

^1^Normal periodic menstrual blood loss = 15–20 pads/cycle. ^2^Abnormal periodic menstrual blood loss = less than 15 and more than 20 pads. ^#^Adjusted for BMI, WHR, and WHtR.

## Data Availability

The data used to support the findings of this study are available from the corresponding author upon request and are archived by the authors, as per the institutional review board policies.

## Abbreviations

BMI: Body mass index
WHR: Waist-hip ratio
WHtR: Waist-height ratio.
